# Cost for the treatment of actinic keratosis on the rise in Australia

**DOI:** 10.12688/f1000research.4671.2

**Published:** 2014-08-20

**Authors:** Eshini Perera, Sean McGuigan, Rodney Sinclair

**Affiliations:** 1 Faculty of Medicine, The University of Melbourne, Melbourne, Parkville, 3052, Australia; 2 Department of Dermatology, Epworth Healthcare, Melbourne, 3121, Australia; 3 Sinclair Dermatology, Melbourne, 3121, Australia

## Abstract

**Objectives: **To report the burden and cost of actinic keratosis (AK) treatment in Australia and to forecast the number of AK treatments and the associated costs to 2020.

**Design and setting:** A retrospective study of data obtained from medicare Australia for AK treated by cryotherapy between 1 January 1994 and 31 December 2012, by year and by state or territory.

**Results: **The total number of AK cryotherapy treatments increased from 247,515 in 1994 to 643,622 in 2012, and we estimate that the number of treatments will increase to 831,952 (95% CI 676,919 to 986,987) by 2020. The total Medicare Benefits Schedule (MBS) benefits paid out for AK in 2012 was $19.6 million and we forecast that this will increase to $24.7 million by 2020 (without inflation).

**Conclusion:** The number of AK cryotherapy treatments increased by 160% between 1994 and 2012. we forecast that the number of treatments will increase by 30% between 2012 and 2020. The rates of non-melanoma skin cancer (NMSC) and AK appear to be increasing at the same rate. During the period 2010 to 2015 AK is anticipated to increase by 17.8% which follows a similar trend to published data that forecasts an increase in NMSC treatments of 22.3%.

## Introduction

Actinic keratosis (AK), is one of the most common ^1^ and most expensive skin diseases to treat. This places a high burden on the Australian population, health care system and government. Up to 60% of Australians in subtropical Queensland over the age of 40 have AK ^2^. AK lesions are rough scaly and generally less than 1 cm in diameter. They are caused by chronic sun exposure and prevented by regular sunscreen use ^3^. Multiple lesions are common. Some lesions regress spontaneously, but others progress to invasive squamous cell carcinoma (SCC). Hypertrophic AK may contain occult foci of SCC ^4^. A systematic review of the natural history of AK estimated the risk of malignant transformation to range from 0% to 0.0075%/lesion/year ^5^. In individuals with a history of non-melanoma skin cancer (NMSC), the risk is up 0.53% per lesion per year ^6^. Organ transplant recipients on systemic immuno-suppressive therapies are at increased risk of malignant transformation ^7^.

Indications for treatment include prophylaxis of SCC, uncertainty of diagnosis, symptomatic relief and to improve cosmetic appearance. Detectable lesions are frequently associated with alterations of the surrounding skin. These surrounding subclinical lesions, or field changes, can be identified histologically and also clinically with the application of topical field therapy for AK ^8^.

Cryosurgery with liquid nitrogen is the most widely used therapy. Clinical response varies with the duration of application, as does morbidity. One study reported cure rates of 39% for freeze times less than 5 seconds, 69% for freeze times between 5 and 25 seconds and 83% for freeze times greater than 25 seconds ^9^.

Medicare Australia is a universal health insurance scheme that reimburses patients for medical fees incurred. Benefits are paid according to the Medicare Benefits Schedule (MBS) with respect to each individual Medicare item number. The item number for treatment of 10 or more solar keratosis by cryosurgery is 30192. There is no item number for treatment of fewer than 10 AK and therefore no reimbursement for cryotherapy treatment of less than 10 AK.

Field therapy is most useful for multiple AK lesions. Agents approved by the Therapeutics Goods Administration for field therapy include topical 5-Fluorouracil (5-FU), diclofenac sodium, inguenol mebutate and photodynamic therapy (PDT). Field therapy is not subsidized by the PBS (Pharmaceutical Benefits Scheme) and does not contribute directly to the cost to Government of treatment of AK in Australia.

There is no current published estimate of the direct cost of AK to the Australian government. We aimed to use Medicare data to report the annual numbers of AK treatments between 1994 and 2012, calculate the direct costs associated with these treatments and predict the numbers and costs from 2013 to 2020.

The risk of AK increases with age and the ageing of the Australian population may increase the burden of AK on the Australian health system.

## Methods

Medicare Australia issues the total number of claims and benefits paid for each Medicare item number according to the Medicare Benefits Schedule (MBS) ^10^. The total number of claims and cost of Medicare benefits for the treatment of 10 or more premalignant lesions with an ablative therapy (MBS item number 30192) was obtained for the period 1994 to 2012 inclusive ^10^.

The historical Medicare benefit payment data were inflated to 2012 Australian dollars, using the Reserve Bank of Australia Inflation calculator ^11^. For each of the SK numbers and costs historical data sets, three Auto-regressive Integrated Moving Average (ARIMA) models ^12^ were fitted and forecasts produced for the years 2013 to 2020. The models were ARIMA(1,1,1), ARIMA(0,1,1) and ARIMA(1,1,0). For each forecast series, the best fitting model was chosen on the basis of the Akaike Information Criterion (AIC) ^13^. For both time series the AIC differences between models were extremely small and the model forecasts very similar.

Total numbers of non-melanoma skin cancer (NMSC) treatment was obtained from a study conducted by Fransen *et al.* ^14^. All statistical analysis was carried out with JMP ^®^ v 9.0.0.

## Results

**Figure 1.**
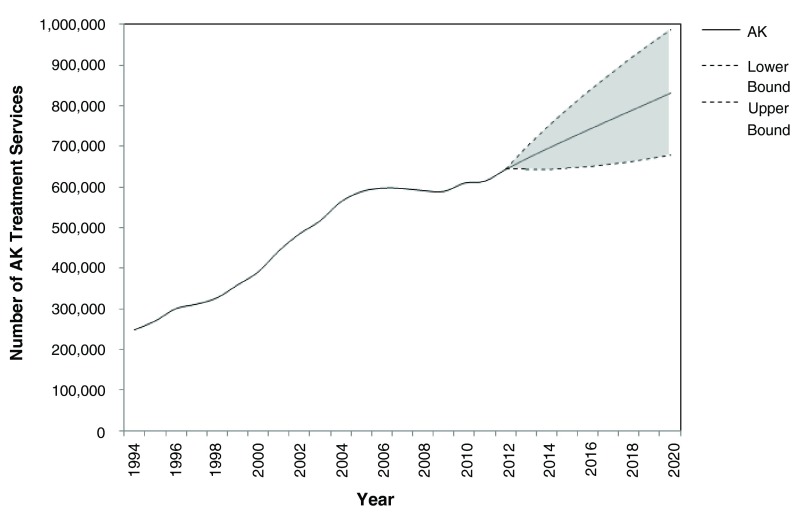
Total number of AK treatments from 1994–2020. Estimated treatment numbers from the period of 1994–2020. Estimated treatment numbers with 95% CI from 2013–2020 is shaded in grey.

**Figure 2.**
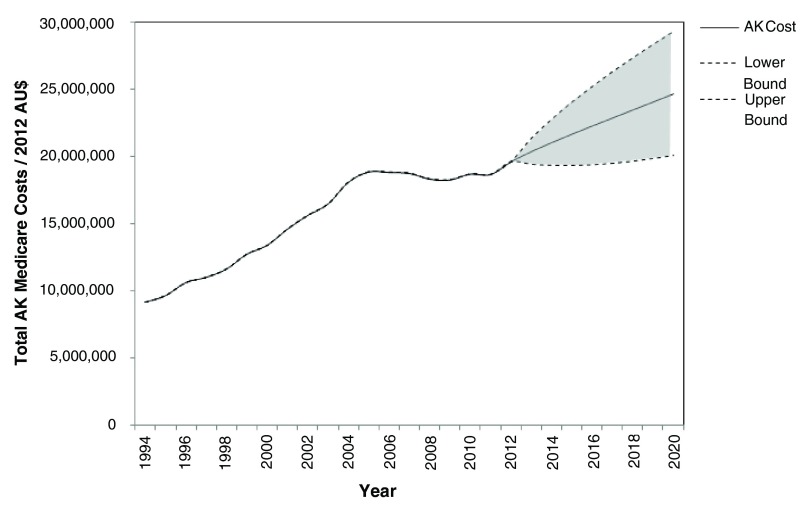
Total cost of AK treatments to Medicare in 2012 Australian dollars from the period of 1994–2020. Estimated costs with 95% CI from 2013–2020 is shaded in grey.

The total number of item 30192 treatment services billed to Medicare in 1994 was 247,515, increasing to 643,622 in 2012. This represents an increase of 160%. Rates remained relatively stable during the period of 2005–2009 and then began increasing again after 2010. It was estimated that the cost of treatments would reach $24.7 million in 2020 for roughly 831,953 treatment services – an increase of 29% over the next 8 years ( Table 1 and Figure 1 and Figure 2).

**Table 1.** Total number of AK treatments, total costs inflated, total costs without inflation and forecasts of AK numbers and costs with upper and lower 95% confidence limits (UCL95 and LCL95).

Year	Number of AK Treatments	Number of AK Treatments LCL95	Number of AK Treatments UCL95	Total cost in non-deflated AU$	Total cost in 2012 AU$	Total cost in 2012 AU$ LCL95	Total cost in 2012 AU$ UCL95
1994	247,515	.	.	5,592,597	9,094,440	.	.
1995	269,252	.	.	6,168,831	9,587,787	.	.
1996	299,863	.	.	6,975,529	10,565,260	.	.
1997	311,091	.	.	7,248,143	10,953,532	.	.
1998	326,626	.	.	7,724,620	11,574,041	.	.
1999	358,525	.	.	8,593,104	12,687,151	.	.
2000	390,535	.	.	9,439,584	13,342,203	.	.
2001	442,486	.	.	10,757,315	14,562,918	.	.
2002	484,561	.	.	11,857,056	15,586,976	.	.
2003	516,626	.	.	12,851,675	16,445,098	.	.
2004	563,261	.	.	14,453,327	18,071,131	.	.
2005	588,726	.	.	15,470,476	18,835,852	.	.
2006	597,756	.	.	15,991,419	18,801,665	.	.
2007	596,683	.	.	16,304,737	18,733,990	.	.
2008	591,497	.	.	16,618,138	18,298,064	.	.
2009	589,286	.	.	16,865,300	18,247,035	.	.
2010	609,820	.	.	17,770,443	18,681,155	.	.
2011	614,843	.	.	18,327,107	18,650,174	.	.
2012	643,622	.	.	19,602,097	19,602,097	.	.
2013	669,766	642,866	696,666	.	20,364,039	19,399,117	21,328,960
2014	694,371	643,980	744,763	.	21,037,418	19,324,949	22,749,887
2015	718,078	645,821	790,335	.	21,669,511	19,309,150	24,029,872
2016	741,260	649,137	833,382	.	22,282,359	19,360,513	25,204,205
2017	764,135	654,047	874,223	.	22,886,235	19,471,254	26,301,216
2018	786,831	660,435	913,227	.	23,485,929	19,630,731	27,341,128
2019	809,422	668,121	950,724	.	24,083,673	19,829,435	28,337,912
2020	831,953	676,919	986,987	.	24,680,509	20,059,777	29,301,241

**Figure 3.**
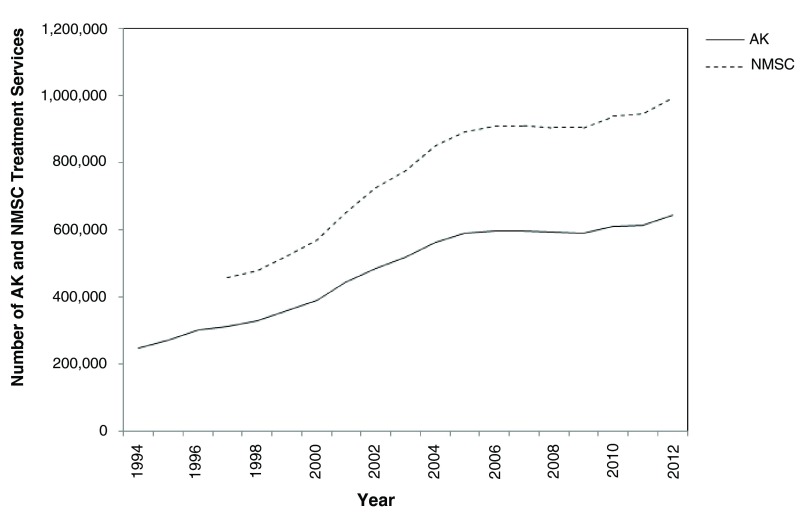
Total number of item 30192 services billed in Australia from 1994 to 2012. Total number of non-melanoma skin cancer treatment for 1997 to 2012 shown in dotted lines.

The total number of AK treatments billed to Medicare showed a similar increase over time to the increase seen in total NMSC treatments over the same period of time ^14^ ( Figure 3). NMSC treatment services continued to rise during 2005–2009 while AK treatment services remained stable.

**Figure 4.**
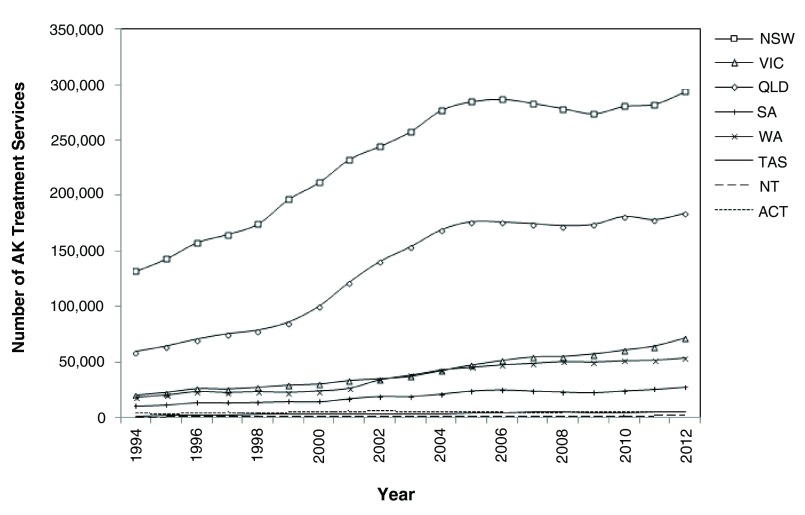
Total number of item 30192 services billed per year for each state from the period of 1994–2012.

The total number of treatment services billed for each of the Australian states and territories is illustrated in Figure 4. Approximately 293,933 treatment services were billed in NSW in 2012 and 184,233 services in Queensland compared to 1,548.

The number of AK treatments per 1,000 head was the highest in New South Wales compared to Queensland ( Table 2). The number of AK treatments per 1,000 people in the Northern Territory was remarkably low, almost comparable to the number of AK treatments seen in Tasmania.

**Table 2.** Number of AK treatments per 1,000 people for each Australian state.

	NSW	VIC	QLD	SA	WA	TAS	NT	ACT
**1994**	21.8	4.7	18.7	7.2	10.5	3.6	3.9	12.6
**1995**	23.3	5.1	19.7	7.9	11.5	3.9	3.8	12.2
**1996**	25.4	5.8	21.3	9.3	13.1	4.8	3.5	14.2
**1997**	26.4	5.7	22.4	9.2	12.4	5.3	3.0	14.7
**1998**	27.6	6.0	23.0	9.4	12.6	6.3	3.4	13.4
**1999**	30.7	6.4	24.7	9.8	12.1	6.7	3.0	14.5
**2000**	32.7	6.5	28.4	9.7	12.6	6.0	3.6	16.4
**2001**	35.5	7.1	33.8	11.5	13.7	6.3	4.4	17.5
**2002**	37.1	7.3	38.1	12.9	17.6	6.5	4.3	18.3
**2003**	38.9	7.7	40.7	12.7	19.4	6.4	3.7	17.1
**2004**	41.6	8.6	43.7	14.1	21.5	7.4	4.3	15.9
**2005**	42.4	9.5	44.6	15.7	22.4	8.3	3.8	14.3
**2006**	42.3	10.1	43.5	16.2	23.0	9.1	3.7	12.9
**2007**	41.2	10.6	42.0	15.3	23.0	10.6	3.0	12.2
**2008**	39.8	10.4	40.5	14.6	22.9	9.9	3.7	13.0
**2009**	38.6	10.6	39.9	14.0	22.2	9.4	4.3	13.2
**2010**	39.1	11.1	40.9	14.9	22.2	9.3	6.0	12.5
**2011**	39.0	11.6	39.5	15.6	21.8	9.9	5.8	12.9
**2012**	40.0	12.6	40.0	16.6	21.8	10.2	6.5	13.8

**Figure 5.**
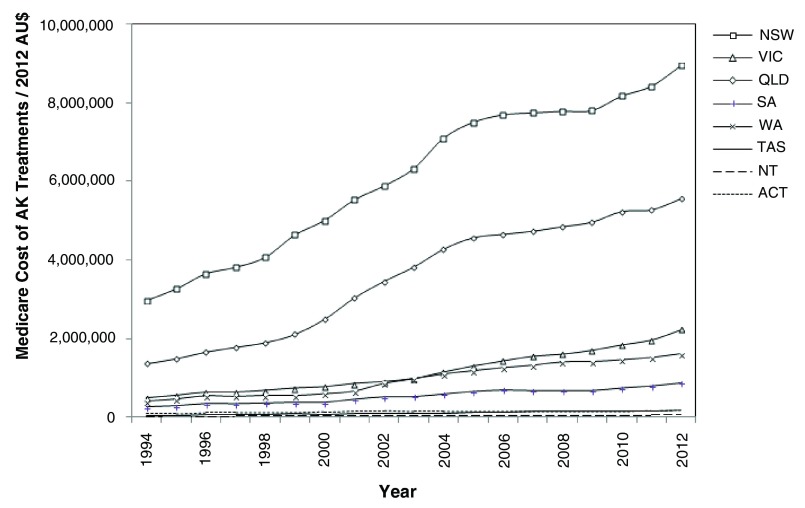
Total benefits paid for item number 30192 for each Australian state and territory (2012 AU$).

The total Medicare benefits paid for the 30192 item number in 1994 was $5.6 million and $19.6 million in 2012. This represents an increase of 250%. NSW and Queensland received the highest amount of benefits at $9 million and $5.6 million respectively) while NT received the lowest amount of Medicare benefits at $49 thousand ( Figure 5).

## Discussion

AK is a chronic disease. Patients with multiple AK require multiple treatment episodes. The number of item 30192 treatments for AK has increased by 160% since 1994 and is predicted to increase by a further 29% between 2012 to 2020. NMSC has similarly been increasing over time. During the period 2010 to 2015 AK is anticipated to increase by 17.8% compared with an increase in NMSC treatments of 22.3% which was predicted by Fransen *et al.* ^14^.

Interestingly, the rates of ablative treatment for premalignant lesions of the skin remained virtually stable during the period 2005 to 2009, whilst the rates for NMSC treatments continued to rise during this period. One possible explanation for the trend in AK billing treatments is the introduction of new field treatment options during this period. The Therapeutic Goods Administration approved PDT with Methyl aminolevulinate (Metvix ^®^) for the treatment of AK in 2003 and Imiquimod (Aldara ^®^) for treatment of AK in 2004.

Topical 5-FU and PDT are funded by the RPBS but not the PBS for the treatment of AK in repatriation members ^15^. The contribution of these agents to the overall cost to Government of treatment of premalignant skin conditions is therefore negligible.

Also notable is the significantly higher number of AK treatment services, provided by NSW compared to Queensland. This is particularly surprising as the number of NMSC treatments recorded in Queensland continued to increase at a faster rate and the number of NMSC treatments was greater than in NSW after 2011 ^10^.

Northern Territory (NT) had the lowest number of AK treatments per 1,000 a figure that was comparable to treatment numbers seen in Tasmania. It was expected that the number of AK treatments per head would have been similar to that of Queensland because of the association between AK and ultraviolet (UV) light and the higher levels of UV exposure in NT. Aboriginal and Torres Strait Islanders make up 30% of the population in the NT ^16^. Although there is limited literature on the incidence of AK in the Indigenous population it is likely that the incidence of AK would be less that the Caucasian population as the incidence rates of NMSC and melanomas are reduced in the Indigenous population ^17^, however this still does not account for the lower than expected treatment numbers. Most of the NT is considered rural or remote under the Australian government’s remoteness classification ^18^ and the AK treatment numbers may reflect the reduced access to healthcare in the NT. Furthermore, the lower number of AK treatments may also reflect the behavioural patterns of health seeking in populations living in remote areas.

One indication for the treatment of AK is to prevent the progression to SCC. Currently there is no up to date incidence data for SCC for the time periods studied in this paper. Further studies could examine if the treatment of AKs correspond to a reduction in cutaneous SCC. These studies would determine if the high cost of treating AK is justified.

There were three major limitations of the study. The data set did not include data on cryotherapy item numbers charged to the Department of Veterans affairs; the data did not include treatment of less than ten lesions; the Medicare item numbers could have included lesions that were treated multiple times; in addition the 30192 item number may include other premalignant conditions of the skin, however we have assumed that AK are the predominant skin lesion. Some AK may clinically resemble SCC and be treated surgically. We have not calculated the costs of skin surgery of AK. Our calculations did not include any patient co-payments costs incurred or the cost of the consultation. Estimates of the number of treatments for other treatment modalities were not available.

The cost to the government and the cost to the community of AK is increasing. We predict that the number of AK treatments provided by the government in 2020 will be 831,953 costing an estimated 24.7 million dollars.

An estimated 60% of SCCs develop from AK and the remaining 40% develop de novo ^19^. Given the high risk of malignant transformation treatment of early lesions and prevention of AK is prudent.

Future research should focus on identifying future AK trends, cost effectiveness of field treatments versus cryotherapy for AK, the association between AK and SCCs and the role of field treatments for clinical and subclinical AKs in preventing SCCs.

## Data availability

The data in this study and the study by Fransen *et al.* can be accessed via Medicare Australia Statistics. http://www.medicareaustralia.gov.au/statistics/mbs_item.shtml. The item number used was 30192; the dates used were 1994–2012.
