# Supplementary material for: Cost for the treatment of actinic keratosis on the rise in Australia
Source: F1000Res. 2014 Aug 20;3:184. Originally published 2014 Aug 5. [Version 2] doi: 10.12688/f1000research.4671.2 (PMC4184293; doi:10.12688/f1000research.4671.2)
Supplement: Peer review history

# Peer review: Referee response for version 2

**DOI:** 10.5256/f1000research.5487.r5918

**Published:** 2014-09-25

**Authors:** Luigi Naldi

**Affiliations:** 1 Department of Dermatology, Centro Studi GISED, Bergamo, Italy

The authors have addressed the critical points I raised.

I have read this submission. I believe that I have an appropriate level of expertise to confirm that it is of an acceptable scientific standard.

# Peer review: Referee response for version 2

**DOI:** 10.5256/f1000research.5487.r5917

**Published:** 2014-09-01

**Authors:** Giuseppe Argenziano

**Affiliations:** 1 Department of Dermatology, Second University of Naples, Naples, Italy

The authors nicely answered the questions posed by the reviewers.

I have read this submission. I believe that I have an appropriate level of expertise to confirm that it is of an acceptable scientific standard.

# Peer review: Referee response for version 1

**DOI:** 10.5256/f1000research.4992.r5704

**Published:** 2014-08-12

**Authors:** Giuseppe Argenziano

**Affiliations:** 1 Department of Dermatology, Second University of Naples, Naples, Italy

This is an interesting study outlining the rising number of treatments of actinic keratoses (AK) and non melanoma skin cancers in Australia. The paper reads well and deals with an important issue.

It would be useful to understand whether the increasing number of treated AKs actually corresponds to a decrease of invasive squamous cell carcinomas. If this is not the case, then the burden and cost of AK treatment may be not completely justified. This issue should be shortly addressed in the discussion.

Please check in the abstract, results section, "...by 202" should read "2020".

I have read this submission. I believe that I have an appropriate level of expertise to confirm that it is of an acceptable scientific standard, however I have significant reservations, as outlined above.

# Response

**Published:** 2014-08-16

**Authors:** Eshini Perera

**Affiliations:** The University of Melbourne, Australia

Thank you kindly for reviewing our article. 

We have amended the article to include your suggestions: 

- Incidence of SCC was briefly mentioned in the discussion. Unfortunately there is no recent data on the incidence of cutaneous SCC. There is also no data which was collected during the time frame of our study.  The increasing number of AK treatments could not be correlated to the number of SCC. 
- The abstract was corrected to read 2020. 

# Peer review: Referee response for version 1

**DOI:** 10.5256/f1000research.4992.r5703

**Published:** 2014-08-06

**Authors:** Luigi Naldi

**Affiliations:** 1 Department of Dermatology, Centro Studi GISED, Bergamo, Italy

This is an interesting study dealing with the number of treatments reimbursed by Medicare for AK in Australia over time, and paralleling those treatments with number of treatments for SCC. As discussed by the Authors, the data presented have several limitations mainly connected with the nature of the claims data available (there is a lack of information on treatments of less than 10 AKs, and a lack of information on treatment modalities other than cryotherapy). In addition, a few points are worth of consideration:

- The Title does not accurately reflect the contents of the paper. I would change it to: "Costs for the treatment of AKs on the rise in Australia"
- The study is restricted to treatment of 10 or more AKs by cryotherapy. Is there any estimate of the number of treatments for modalities other than cryotherapy (including field therapy)?
- Is there any indication that some lesions were not treated at all?
- Were repeated treatments of the same lesions counted separately or did they simply sum up to the total?
- Introduction, 5th paragraph: what does "PBS" stand for?
- Methods, 3rd paragraph: as far as I understand the meaning, the reference should be to Figure 3 and not to Figure 1.
- Discussion, penultimate paragraph: "treatment of early treatment and prevention of AK is prudent" should read "treatment of early lesions and prevention of AK is prudent"

I have read this submission. I believe that I have an appropriate level of expertise to confirm that it is of an acceptable scientific standard, however I have significant reservations, as outlined above.

# Response

**Published:** 2014-08-16

**Authors:** Eshini Perera

**Affiliations:** The University of Melbourne, Australia

Thank you for reviewing our article. 

We have made the following changes to our article, as per your recommendation:

- The title was amended to reflect the content of the paper.
- There are no estimates for the number of treatments for modalities other than cryotherapy. This was mentioned in the article. 
- It is possible that some lesions were not treated and this was mentioned in the article. 
- It is possible that some lesions could have been treated twice and this was mentioned in the article. 
- The 3rd paragraph in the methods section was amended so that the  meaning reflected what was seen in Figure 1. 
- The error in the discussion paragraph was corrected. 

We thank you for your suggestions.
